# Dr. Gunning S. Bedford’s Works in Foreign Countries

**Published:** 1866-12

**Authors:** 


### Dr. Gunning S. Bedford's Works in Foreign Countries.

The two works by this distinguished American author have been received with great favor by the profession both of his own and foreign countries. The one on Diseases of Women and Children is now in its eighth edition, and receives many notices from foreign journals similar to the following, which must be very gratifying to an American author:

"Dr. Bedford's book is worthy of its author, a credit to his country, and a valuable mine of ihstraction to the profession at large. We are quite sure that it will he a welcome addition to professional libraries in Great Britain as well as America."—*Brit. and For. Med. Chir. Review.*

"We were actually fascinated into reading this entire volume, and have done so most attentively; nor have we ever read a book with more pleasure and profit. There is not a disease connected with infancy or the female system which ts not most ably discussed in this excellent work."—*Dublin Quarterly Journal of Medical Science.*

"A careful perusal of Dr. Bedford's book has led us to believe that its value will continue to be acknowledged, and the author recognised as a most able and acute practitioner of medicine. The work is of the most practical character; everything is made to tend towards the relief and treatment of disease, and remarkable skill is shown in quickly arriving at an accurate diagnosis. To get at once to the point is the pervading characteristic of the author's teachinge. We cordially recommend it to all practitioners and students of medicine." *London Lancet.*

"It is to be regretted that we have not more such books in Great Britain."—*London Medical Times and Gazette.*

"The style of the author is very graphic. The book not only proves Dr. Bedford to be a sound physician and an excellent clinical teacher, but it also affords evidence of an extensive acquaintance on his part with the literature of his subject on this side of the Atlantic."—*London British Medical Journal.*

Similar notices of the work on Obstetrics also appear in foreign journals. The terms of approval are full and hearty.

"It was with considerable interest that we opened the imposing volume which Dr. Bedford has given to the world. It has cost its author many an hour of thought and toil, which would otherwise, perhaps, have constituted the leisure of a busy life. The fact particularly has struck us much in reading this book—the author seems to think no point too trivial to notice. With great good sense he instructs his readers in matters which are too often discussed in a few words. We have read the work with great pleasure, and as a practical guide is a truly excellent one—perhaps, in this respect, it is unsurpassed."—*Glasgow Med. Journal.*

"The work of Dr. Bedford is, as its title implies, a complete Systematic and Practical Treatise upon Obstetrics, brought up to the existing state of the science, and embracing the anatomy, physiology, signs and diseases of pregnancy, parturition, and child-bed. All these subjects, with the physiological disquisitions arising out of some of them, are discussed. Assuredly, so comprehensive a course was never, perhaps, before given. The volume is evidently the result of much labor and research, and contains a vast deal of information, and that of a recent kind, upon nearly all the subjects connected directly or indirectly with midwifery." *London British and Foreign Medico-Chirurgical Review.*

American authors may not reasonably expect anything more than they deserve from foreign reviewers. Dr. Bedford may consider himself "well used," for they have all fully recommended both of the works he has recently given to the world.

